# Contribution of Arab countries to breast cancer research: comparison with non-Arab Middle Eastern countries

**DOI:** 10.1186/s12905-015-0184-3

**Published:** 2015-03-14

**Authors:** Waleed M Sweileh, Sa’ed H Zyoud, Samah W Al-Jabi, Ansam F Sawalha

**Affiliations:** 1 Department of Pharmacology and Toxicology, College of Medicine and Health Sciences, An-Najah National University, Nablus, Palestine; 2 Department of Clinical and Community Pharmacy, College of Medicine and Health Sciences, An-Najah National University, Nablus, Palestine

**Keywords:** Bibliometric, Breast cancer, Arab world, Middle Eastern Region, ISI Web of Science

## Abstract

**Background:**

Breast cancer is one of the most common types of cancers affecting women worldwide. The main objective of this study was to assess and compare research activity in breast cancer in Arab countries with non-Arab Middle Eastern countries.

**Methods:**

Publications about “breast cancer” as a research topic were retrieved using the ISI Web of Science database. Analysis was confined to original research and review articles. Research productivity was assessed by assessing number of publications and time trend of these publications, names of journals, citation analysis, top 10 active institutions as well as country contribution to breast cancer research. The quantity and quality of publications from Arab countries in addition to 3 other Middle East countries (Turkey, Iran and Israel) were assessed and compared using the *h*-index tool.

**Results:**

A total of 1658 original research and review articles about “breast cancer” were published from Arab countries. Annual research productivity from Arab countries in the field of “breast cancer” was negligible but showed a significant increase in the last decade. Retrieved documents had relatively high citation parameters as measured by h-index of 61 and average citations of 17.46 per document. The highest research productivity was from Egypt with a total publication of 582 (35.10%). Cairo University with a total of 149 (8.99%) publications had the highest research productivity among institutions in Arab world. Forty four documents (2.65%) of breast cancer documents were published in *Saudi Medical Journal.* Arab researchers collaborated mostly with researchers from the United States of America (305; 18.40%) in breast cancer research. Compared with other non-Arab Middle Eastern countries, Arab countries had higher research productivity than some countries and lower than others, particularly Israel.

**Conclusions:**

The present data reveals a good contribution of some Arab countries to the field of “breast cancer” research. There is a gap between Arab countries and Israel in the quality of breast cancer research.

## Background

Breast cancer is one of the most common types of cancers among women in the Arab world [1]. Research in general and in breast cancer in particular is important for each country in order to understand the cultural and genetic differences among different people in different geographical areas. Therefore, assessing research about breast cancer from Arab countries has an important impact on the future policies in the fight against such an emotionally disturbing type of cancer. In addition, no doubt that high research activity in breast cancer can reflect positively on screening programs, awareness, epidemiological data and clinical practice. Assessing research activity in any subject in any geographical region is not an easy task given the diversity of academic databases and indexing methods of such databases. One of the most commonly utilized methods to assess research activity is known as bibliometric methods which have been applied to various medical subjects in different countries [2-8]. No doubt that the Arab world have witnessed great progress in the past few decades in medical education and medical practice including establishing high standards private hospitals and private medical colleges. Furthermore, many governments in the Arab world, particularly in the Arab Gulf, have financially endorsed the development of medical research and practice to serve the general public health of their people. The Arab world has an overall population of around 380 million and extends over a large geographical area in northern Africa and Middle East. Our research group have previously published several bibliometric studies in different medical disciplines like osteoporosis, diabetes, toxicology and others [9-11]. In this study, our research group is trying to shed light on a very important health topic pertaining to women’s health and public health as well which is breast cancer research activity in Arab countries. The objective of this study was to assess publication trend in Arab countries in breast cancer and compare it with non-Arab Middle Eastern countries. Such study will give an insight into research activity and will encourage those in the field of breast cancer.

## Methods

The methodology as well as the tool used in this study were discussed in previous publications by the same group [11,12]. The tool used was ISI Web of Science (WoS) [13]. The method used in this study is similar to the method used by the authors in their previous publications [11,14,15]. For the purpose of this study, WoS is preferred over PubMed because PubMed does not allow for citation analysis. Similarly, WoS is preferred over Scopus because WoS includes the most influential and prestigious journals in all medical fields. All Arab countries were used as country keys followed by “breast cancer” phrase as a topic. We focused on the phrase “breast cancer” as search topic (TS) and excluded any other associated phrases like “mammary ducts” because we are interested in breast cancer per se rather than associated cancers. Because the WoS database does not recognize Palestine as an independent state yet, search for breast cancer documents from Palestine was carried out using separate search keys. The results from 21 Arab countries and those from Palestine were combined and the resultant data were analyzed. Only original research articles and review articles were included in the analysis. Furthermore, analysis was made up to year 2012 while 2013 and 2014 years were excluded because they are still open for new journal issues. The standard competition ranking (SCR) was used to present the top 10 ranking data while journal’s impact factors (IF) were evaluated using the Journal Citation Report (JCR; Web of Science) 2013 science edition by Thomson Reuters (New York, NY, USA).

## Results

The total number of worldwide publications in breast cancer retrieved from Arab countries was 1658 publications while worldwide number was 206,710. Therefore, “breast cancer” research output from Arab countries represents 0.8% of the global research productivity in “breast cancer”. The first article about breast cancer co-authored by an Arab researcher was published in 1977 in *Cancer* Journal and the title of the article was: “Clinical and prognostic features of a rapidly progressing breast-cancer in Tunisia”.

**Figure 1:**
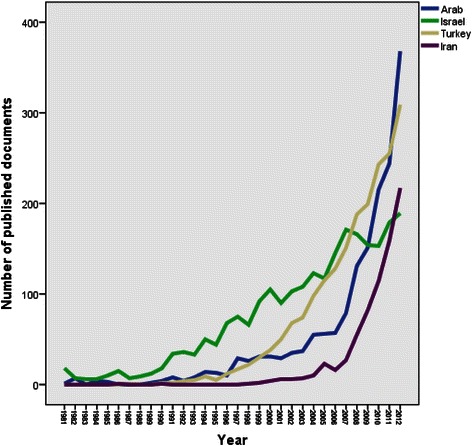
**Annual growth of breast cancer research output from Arab countries, Turkey, Israel and Iran.** Earlier data were not shown in the figure because it is almost approaching zero for most Arab countries.

The annual number of publications from Arab countries indicated that “breast cancer” research output remained low until mid-1990s and showed a significant increase in the last three years. Approximately 50% of breast cancer documents from Arab countries were published in 2010, 2011 and 2012. Figure 1 shows the annual growth of breast cancer research documents from Arab countries. When retrieved data were analyzed by country, Egypt (582; 35.10%) had the highest research output followed by Kingdom of Saudi Arabia (372; 22.44%) and Tunisia (172; 10.37%). No data related to breast cancer was found from Somalia, Djibouti, Mauritania and Comoros (Table 1). The language of most breast cancer documents published from Arab countries was English (1589; 95.84%) while the remaining documents were published in French language (69; 4.16%). One hundred and ninety three (11.64%) documents were open access while the remaining (88.36%) were not open access. Countries whose researchers collaborated most in breast cancer research with investigators in the Arab world include the United States of America (USA); (305; 18.40%) followed by France (139; 8.38%) and England (132; 7.96%).

**Table 1:** **Contribution of each Arab country to the 1658 breast cancer published documents**

Country	Number of documents N (%) = 1658 (100%)*	*h*index
Egypt	582 (35.10)	43
Kingdom of Saudi Arabia	372 (22.44)	31
Tunisia	172 (10.37)	26
Kuwait	121 (7.30)	21
Lebanon	120 (7.24)	23
United Arab Emirates	89 (5.37)	22
Jordan	79 (4.76)	18
Morocco	73 (4.40)	15
Qatar	32 (1.93)	11
Algeria	27 (1.63)	9
Sudan	21 (1.28)	6
Iraq	21 (1.28)	7
Libya	20 (1.21)	6
Syria	18 (1.09)	8
Yemen	17 (1.03)	7
Oman	13 (0.78)	7
Palestine	10 (0.52)	6
Bahrain	7 (0.42)	3
Somalia	0 (0.0)	0
Comoros	0 (0.0)	0
Djibouti	0 (0.0)	0
Mauritania	0 (0.0)	0

*total exceeds 100% because of overlap in some documents among more than one Arab country.

The total number of citations for breast cancer documents from the Arab world, at the time of data analysis, was 28,953 with an average citation of 17.46 per document. Of the 1658 documents considered for the *h*-index, 61 had been cited at least 61 times at the time of data analysis. When stratified by country, the *h* index was highest for breast cancer documents published from Egypt (*h*-index = 43) followed by those published from KSA (*h*-index =31) and Tunisia (*h*-index =26). Of the 1658 breast cancer documents published from Arab countries, there were 660 (39.81%) documents in the oncology research area, 185 (11.16%) in the pharmacology/pharmacy research areas, and 128 (7.72%) documents in Biochemistry/Molecular Biology. Other research areas found in published documents from the Arab countries are shown in Table 2.

**Table 2:** **Research areas of the 1658 breast cancer documents published from the 22 Arab countries**

SCR^a^	Research area	Number (%) N = 1658 (100%)
**1st**	Oncology	660 (39.81)
**2nd**	Pharmacology Pharmacy	185 (11.16)
**3rd**	Biochemistry Molecular Biology	128 (7.72)
**4th**	Pathology	103 (6.21)
**5th**	General Internal Medicine	101 (6.09)
**6th**	Chemistry	94 (5.67)
**6th**	Obstetrics Gynecology	94 (5.67)
**8th**	Cell Biology	89 (5.37)
**9th**	Radiology Nuclear Medicine Medical Imaging	74 (5.49)
**10th**	Surgery	54 (3.26)

*Abbreviation:* SCR = Standard Competition Ranking.

^a^Equal research areas have the same ranking number, and then a gap is left in the ranking numbers.

Table 3 lists the top 10 journals in which documents in breast cancer were published from Arab countries. Forty four (2.65%) breast cancer documents from Arab countries were published in *Saudi Medical Journal* which is a non-oncology journal. Of the top 10 journals in which breast cancer documents were published from the Arab countries, only 1 journal (*International Journal of cancer Research*) had an impact factor > 5. Table 4 shows the top 10 most productive Arabic institutions in breast cancer. The most productive institution was Cairo University (149; 8.99%) followed by King Saud University (117; 7.06%). Eight institutions in the top 10 list are academic institutions affiliated with teaching hospitals that have oncology departments.

**Table 3:** **Top 10 journals in which breast cancer documents from the 22 Arab countries were published**

SCR^a^	Journal	Number (%) N = 1456	IF^b^
**1st**	*Saudi Medical Journal*	44 (2.65)	0.554
**2nd**	*Asian Pacific Journal of Cancer Prevention*	41 (2.47)	1.50
**2nd**	*Breast Cancer Research and Treatment*	41 (2.47)	4.19
**4th**	*Anticancer Research*	38 (2.29)	1.872
**5th**	*Breast*	25 (1.51)	2.581
**6th**	*European Journal of Medicinal Chemistry*	24 (1.45)	3.432
**6th**	*Medical Oncology*	24 (1.45)	2.058
**9th**	*Life Science Journal Acta Zhengzhou University Overseas Edition*	22 (1.33)	0.165
**10th**	*Cancer*	20 (1.21)	4.901
**10th**	*International Journal of Cancer*	20 (1.21)	5.007

*Abbreviations:* SCR = Standard Competition Ranking; NA = not available; IF = impact factor.

^a^Equal journals have the same ranking number, and then a gap is left in the ranking numbers.

^b^The impact factor was reported according to Institute for Scientific Information (ISI) journal citation reports (JCR) 2012.

**Table 4:** **Top 10 active institutions in breast cancer research in Arab countries**

SCR	Institution	Number (%) N = 1658	Country
**1st**	Cairo University	149 (8.99)	Egypt
**2nd**	King Saud University	117 (7.06)	KSA
**3rd**	King Faisal Specialist Hospital Research Center	115 (6.94)	KSA
**4th**	Kuwait University	104 (6.27)	Kuwait
**5th**	American University of Beirut	83 (5.01)	Lebanon
**6th**	Alexandria University	56 (3.38)	Egypt
**7th**	Mansoura University	53 (3.20)	Egypt
**8th**	Ain Shams University	47 (2.84)	Egypt
**9th**	Assiut University	46 (2.97)	Egypt
**10th**	National Research Center	45 (2.71)	Egypt

*Abbreviations:* SCR = Standard Competition Ranking; KSA = Kingdom of Saudi Arabia.

**Table 5:** **Breast cancer research output from Arab countries compared with that from Turkey, Israel, and Iran**

Data/Country	22 Arab countries	Turkey	Israel	Iran
**Number of population in millions**	380	60	8	80
**Number of published research articles and review articles**	1658	2025	2440	731
**Total number of citations**	28,953	22,438	96,323	7807
**Average citation per document**	17.46	11.08	39.48	10.68
**h-index**	61	56	128	37
**Country with the highest collaboration (%)**	USA 18.40%	USA 8.64%	USA 26.11%	USA 6.98%
**Journal with the highest publication (N); (IF)**	*Saudi Medical Journal* (44) **IF = 0.554**	*Asian Pacific Journal of Cancer Prevention* (78) **IF = 1.50**	*Cancer Research* (87) **IF = 9.28**	*Asian Pacific Journal of Cancer Prevention* (64) **IF = 1.50**

Compared with other non-Arab countries in the Middle East, the research productivity from the Arab countries was lesser than that from Israel and Turkey but higher than that from Iran (Figure 1). The *h* index of breast cancer documents published from Arab countries was higher than that of Turkey and Iran but was much lower than that from Israel. The journal with the highest number of breast cancer documents published from Arab countries was Saudi Medical Journal (IF of 0.55); that from Turkey and Iran was *Asian Pacific Journal of Cancer Prevention* (IF = 1.50), while that from Israel was *Journal of Cancer Research* (IF = 9.28); (Table 5). The annual growth of breast cancer research output from Arab countries, Israel, Turkey and Iran is shown in Figure 1. Breast cancer research in Israel started as early as 1973 and showed a continuous momentum since then. However, breast cancer research in Arab countries, Turkey and Iran started breast cancer research after 1977 and showed great fluctuations up until mid-1990s. Regarding research interests, biochemistry/ genetics and cell biology constituted approximately 25% of breast cancer documents from Israel but less than 10% in Arab countries or Turkey or Iran. Finally, it should be noted that of the documents published from Israel, there were 16 documents about breast cancer in Israeli Arab women, either in comparison with Jewish women or being investigated as a separate cultural group.

## Discussion

In this study, we assessed the research activity about breast cancer from Arab countries. We used the ISI web of science database as a method to assess research activity. Up to the author’s best knowledge, this is the first bibliometric study to assess breast cancer research activity from Arab world. Bibliometric studies that assessed global breast cancer research productivity has been previously published but with no detailed emphasis on Arab region [16,17].

The result indicated that 1658 documents in breast cancer were published from Arab countries up to year 2012. This number is an important piece of information for future analysis and for health policy makers. It should be emphasized that some journals in which Arab researchers have published about breast cancer are not indexed in ISI web of science. For example, articles pertaining to breast cancer that were published in Journal of the Egyptian National Cancer Institute or Eastern Mediterranean Health Journal were not considered in the analysis since these journals are not listed in the WoS. Therefore, the result obtained might be lesser than the actual research activity in Arab countries. Despite that, our study reflects a close approximation of breast cancer research activity in the Arab world and that is present in international and reputable journals. The ISI of Web of Science gives a powerful and attractive citation analysis for bibliometric studies. Furthermore, the ISI Web of Science database reflects publications in journals which have scientific and international impact as well as large audience and readers in the field of breast cancer.

Breast cancer is one of the most emotionally devastating types of cancers for women all over the world. One of the best methods to combat breast cancer and improve the quality of life of women in Arab world is to do breast cancer screening surveys. Unfortunately, it has been reported that breast cancer screening participation rates are low among women in the Arab world [18]. Efforts to combat breast cancer at the global and regional levels should be collective and all those in field need to contribute in this fight against breast cancer at all levels. Research is an important aspect and contribution that can help in combating breast cancer globally and at the regional level. The importance of regional research about breast cancer stems from the fact that cultural and genetic variations might affect breast cancer disease profile among women in different regions and countries.

Our results showed that breast cancer research was neglected in most Arab countries during the 1970s and 1980s. Despite the valuable contribution of Arabs to medicine throughout history [19], reports indicated that medical research activity in Arab world is still lagging behind compared to non-Arab countries in the region like Israel, Turkey or Iran [9,20-24]. Until the time of writing this manuscript, breast cancer research activity is considered low in many Arab countries including those with good economy like Bahrain. On the other hand, breast cancer research in Egypt and KSA showed a steep rise in the past decade. Actually, some of the research activity in Egypt and KSA was governmentally funded suggesting a national policy to promote cancer research as one preventive strategy to combat this devastating type of cancer. Our findings regarding research activity from Egypt and KSA might not be surprising given that previous research indicated that Egypt and KSA have high biomedical research output [24,25]. Low research activity in some Arab countries is a multi-faceted problem that could be attributed at least in part to, inadequate research infra- structure in many cancer institutions in these countries, the modest governmental and non-governmental funding, in addition to a poor cooperation between industry and academia. Governments in the Arab world need to consider these factors if they wish to improve the status of their scientific research [24,26-28].

Our study indicated that the average citation per breast cancer document from Arab world was 17.46. This is relatively a high number and suggests that there is a growing interest in breast cancer from the Arab world and that competition in this field is growing as well. Highly cited articles positively contribute to the *h*-index of the individual author and to the institution and country [29-32]. The citation is a key indicator of research quality [33]. The h-index simultaneously measure the quality and quantity of scientific output. However, *h*-index measured using different databases can give different values and therefore each database has pros and cons when measuring the h-index [34-36]. Criticisms have also been addressed to the use of *h*-index as a marker of publication quality and citation. For example, the h-index does not consider the context of citations, the number of authors in the document, and gives equal values for book citation and research citation. Therefore, the *h*-index has lesser predictive accuracy and precision than mean citations per paper, although this is controversial [37,38].

In our study, the *h*-index of breast cancer documents published from Israel was higher than that for breast cancer documents published from Arab countries or Iran or Turkey. One possible reason for this is the impact factor of journals in which Israeli researchers publish their breast cancer research. Israeli researchers with high quality research tend to publish their research in high impact factor journals like *Cancer Research Journal.* Such publications in high impact journals might have increased the citations for Israeli publications in the field of breast cancer [39,40]. When submitting a manuscript for publications, authors are usually inclined to submit to journals that are publication free, follow open access policy, preferably have a high impact factor, and above all have a short editorial and revision time [41,42]. Researchers from Arab world have preferred publishing breast cancer research in *Saudi medical Journal.* This medical journal poses no publication fee and represents an open forum for medical research discussions in the Arab world.

Our results showed that authors from Arab and non-Arab countries like Israel, Turkey and Iran collaborated mainly with authors from the USA in breast cancer research. International collaboration is beveled to increase the quantity and quality of research activity [43-45]. Breast cancer research in Israel was evidently better than that from Arab countries in quantitative and qualitative terms. One possible reason for this is the intensive collaboration between Israeli and the USA researchers in the field of breast cancer. One explanation for this intensive collaboration between Israeli and the USA researchers is the fact that the status of cancer research in Israel is parallel to that in the USA. Collaboration in research activity can increase the visibility, quantity and quality of research output from any particular country or institution [46-50].

Our study has points of strength and points of weaknesses. This is the first study in the Arab world to investigate and assess breast cancer research activity. The study showed that only few Arab countries, particularly Egypt and KSA had good research output in breast cancer while other Arab countries had limited research activity in this field. Of course, this study does not mean to negatively criticize research activity in any particular country, rather it meant to draw attention and encourage researchers in this field. Finally, it should be mentioned that our study has few limitations which are the same as those of previously published bibliometric studies [51-53].

## Conclusion

The present data reveals a good late momentum for breast cancer research activity from the Arab world. However, the quantity and quality of breast cancer research was low for some Arab countries. Governmental funding and survey programs are important to increase the volume of data available for research in Arab countries. No doubt that researchers in Arab countries need to invest more in the molecular biology and genetics of breast cancer among Arab in order to bridge the gap in this field of research with Israeli researchers. Higher education institutions and research centers in Arab countries should build new bridges of collaboration with their international counterparts to promote breast cancer research in Arab countries.

## Abbreviations

ISI: Institute for Scientific Information
KSA: Kingdom of Saudi Arabia
USA: United States of America
JCR: Journal Citation Report
SCR: Standard Competition Ranking
IFs: Impact factors
WoS: Web of Science
